# The development and malleability of executive control abilities

**DOI:** 10.3389/fnbeh.2014.00221

**Published:** 2014-06-24

**Authors:** Nina S. Hsu, Jared M. Novick, Susanne M. Jaeggi

**Affiliations:** ^1^Center for Advanced Study of Language, University of MarylandCollege Park, MD, USA; ^2^Department of Psychology, University of MarylandCollege Park, MD, USA; ^3^Program in Neuroscience and Cognitive Science, University of MarylandCollege Park, MD, USA; ^4^Department of Hearing and Speech Sciences, University of MarylandCollege Park, MD, USA; ^5^School of Education, University of California, IrvineIrvine, CA, USA

**Keywords:** executive function, neuroimaging, fMRI, working memory, training, interventions, connectivity analysis

## Abstract

Executive control (EC) generally refers to the regulation of mental activity. It plays a crucial role in complex cognition, and EC skills predict high-level abilities including language processing, memory, and problem solving, as well as practically relevant outcomes such as scholastic achievement. EC develops relatively late in ontogeny, and many sub-groups of developmental populations demonstrate an exaggeratedly poor ability to control cognition even alongside the normal protracted growth of EC skills. Given the value of EC to human performance, researchers have sought means to improve it through targeted training; indeed, accumulating evidence suggests that regulatory processes are malleable through experience and practice. Nonetheless, there is a need to understand both whether specific populations might particularly benefit from training, and what cortical mechanisms engage during performance of the tasks used in the training protocols. This contribution has two parts: in Part I, we review EC development and intervention work in select populations. Although promising, the mixed results in this early field make it difficult to draw strong conclusions. To guide future studies, in Part II, we discuss training studies that have included a neuroimaging component – a relatively new enterprise that also has not yet yielded a consistent pattern of results post-training, preventing broad conclusions. We therefore suggest that recent developments in neuroimaging (e.g., multivariate and connectivity approaches) may be useful to advance our understanding of the neural mechanisms underlying the malleability of EC and brain plasticity. In conjunction with behavioral data, these methods may further inform our understanding of the brain–behavior relationship and the extent to which EC is dynamic and malleable, guiding the development of future, targeted interventions to promote executive functioning in both healthy and atypical populations.

## INTRODUCTION: THE IMPORTANCE OF EXECUTIVE CONTROL

Most of the time, people’s rich experiences enable them to navigate the world using a set of habitual (or well-learned) behaviors. Situations sometimes arise, however, that necessitate on-the-fly changes to these routines. For example, an unexpected road closure can require a shift in the usual route one takes to work. In this and similar circumstances, people must deploy executive control (EC) to countermand dominant thoughts and behaviors in favor of irregular actions. In general, EC refers to the guided regulation of thought and action to match internal or task-relevant goals, particularly in novel situations. Importantly though, EC is not a unitary process but refers to a constellation of separable components that collectively work to guide goal-directed behavior (Norman and Shallice, 1986; Botvinick et al., 2001; Miller and Cohen, 2001). Some components – appearing in different theoretical frameworks under a variety of guises – include a control system to manipulate information within short-term memory (Baddeley and Hitch, 1974), and overlapping but separable processes such as self-regulation and -awareness, task-switching, updating, and response inhibition (Miyake, 2000; Barkley, 2001; Friedman and Miyake, 2004). It is thought that these components can operate over a wide variety of domains including working memory (WM) and language processing (Smith and Jonides, 1999; Novick et al., 2005; Thompson-Schill et al., 2005; Badre and Wagner, 2007). In this review, we refer to EC in a broad sense to include attentional control, cognitive control, and self-regulatory behavior (Jonides et al., 1998; Miller and Cohen, 2001; Thompson-Schill et al., 2005).

**FIGURE 1:**
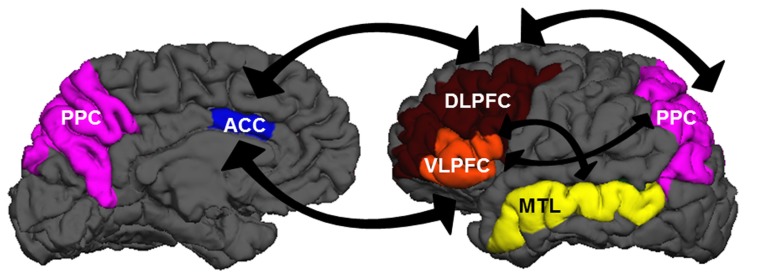
**A wide network of brain regions is recruited during EC processes**. The panels above show medial (left) and lateral (right) views of the left hemisphere. Regions in the EC network include dorsolateral prefrontal cortex (DLPFC: brown), ventrolateral prefrontal cortex (VLPFC: orange), anterior cingulate cortex (ACC: blue), posterior parietal cortex (PPC: purple), and medial temporal lobe structures (MTL: yellow). There is extensive cross-talk between these regions and with other regions sub-serving perceptual, motor, and affective/emotional functions.

Although researchers generally agree that the neurobiological systems underlying EC involve the prefrontal cortex (PFC), the precise manner in which regions within the PFC support cognitive components of EC is still debated (**Figure 1**). Lateral regions of PFC may become engaged under multiple EC demands in a variety of tasks (Thompson-Schill et al., 1997; Duncan and Owen, 2000; Jonides et al., 2008). The anterior cingulate cortex (ACC), a medial frontal region, is thought to be involved in conflict monitoring, detecting situations that may be incompatible with current task goals or demands (e.g., Botvinick et al., 2001), which then signal adjustments in behavior to lateral PFC regions (Kerns et al., 2004). Altogether, these cortical regions receive multiple inputs from and project outputs to virtually all perceptual and motor cortical areas, affective/emotional networks, and subcortical structures. These connections provide an ideal infrastructure for integrating multiple sources of information and guiding subsequent thoughts and actions in a top-down fashion (e.g., Amso and Casey, 2006). We should note here: neural regions that support EC processes are not necessarily restricted to PFC. The capacity to focus attention may be constrained by parietal as well as frontal mechanisms, with increased executive demands modulating activity in posterior parietal (PPC) and dorsolateral frontal cortices (e.g., Wager and Smith, 2003). Additionally, medial temporal lobe (MTL) structures may be recruited in task-specific situations that require the establishment of novel relations (Ranganath and Blumenfeld, 2005).

A traditional assumption has been that while EC and other elements of higher cognition develop rapidly through childhood and adolescence, they remain relatively fixed throughout adulthood. However, recent evidence has challenged this assumption: first, EC processes may follow a non-linear developmental trajectory, in which the maturation of these abilities is subject to changes in brain morphology (Taylor et al., 2013). These processes – driven in part by both heritable and environmental factors – may in fact be subject to experience-dependent plasticity throughout the lifespan (Gray and Thompson, 2004; Bialystok et al., 2006; Neville et al., 2013; Rebok et al., 2014). In particular, interest has grown in lab-based interventions targeting EC components with the aim of also improving performance on other tasks that rely on similar processes (Morrison and Chein, 2010; Jaeggi et al., 2011; Hussey and Novick, 2012).

The importance of intervention work targeting EC is underscored by the accumulating evidence that EC operates across other cognitive domains, including WM (e.g, Smith and Jonides, 1999). In particular, WM abilities predict a wide range of practical outcomes that are important in everyday life, including reading comprehension and mathematical skills (de Jonge and de Jong, 1996; Passolunghi and Siegel, 2001; Gathercole et al., 2006), planning and problem solving (Shah and Miyake, 1999), language processing (Novick et al., 2005), self-regulatory behavior (Hofmann et al., 2012), and scholastic achievement (Duncan et al., 2007; Alloway and Alloway, 2010). Further, deficits in EC and WM abilities are prevalent in a host of clinical syndromes and psychopathologies including attention deficit hyperactivity disorder (ADHD; Shah et al., 2012), depression (Christopher and MacDonald, 2005), and addiction (Khurana et al., 2013); it is also among the core domains susceptible to age-related decline (Braver and West, 2007). In sum, EC is relevant to common assessments of achievement, is sensitive to developmental changes, and many populations suffer from deficits in EC. Given the importance of these abilities to daily life, there has been growing research interest in training paradigms targeting EC abilities with the aim of boosting the improvement (and forestalling the decline) of other complex cognitive skills that rely on EC. Despite the growing literature, there is still a need to understand for whom and at what stages of development EC interventions work best, and also to understand better the cortical mechanisms that underlie training and transfer effects.

The purpose of this paper is to evaluate the current state of the intervention field focusing on selected developmental populations that demonstrate the potential for greater brain plasticity. Because these populations typically demonstrate exaggeratedly poor abilities to control cognition, they may be candidates who are most amenable to receiving maximal transfer benefits. We will review the current work on the neural correlates of training, and suggest methodological directions that may inform a better understanding of the neural mechanisms underlying EC performance. We will outline the current literature on interventions targeting EC (though we note that occasionally different terminology is used to refer to these interventions, including attentional control, and WM), placing a particular emphasis on at-risk populations (e.g., ADHD and low-socioeconomic status; low-SES) that demonstrate performance differences in these domains relative to healthy, adult groups. Such variation suggests that these special groups might be candidates for interventions that seek to improve EC abilities through experience-based plasticity (i.e., an interaction of biological and environmental factors that results in structural and functional changes in brain morphology, as well as concomitant cognitive changes). Much of the current knowledge concerning the malleability of EC comes from the growing body of literature demonstrating that EC can be trained in healthy populations through extensive practice, and that improved performance over the course of training can generalize to novel tasks that were not part of the training regimen. Across a range of different EC tasks, examples of observed transfer benefits include tasks tapping fluid reasoning (Jaeggi et al., 2008), WM updating (Dahlin et al., 2008), task switching (Karbach and Kray, 2009), visual search (Kundu et al., 2013), and language processing (Novick et al., 2014). Although there is little debate as to whether performance on EC tasks can be improved, there is considerable debate around the transferability to novel, untrained tasks (e.g., Redick et al., 2012; Sprenger et al., 2013; Thompson et al., 2013). Transferability is critical to adjudicating between whether EC skills *per se* are affected during training versus whether people simply develop task-specific strategies. One potential explanation for these mixed findings may lie in individual differences in the training-transfer relationship, as well as in the strength of the linking assumptions that tie training and transfer tasks together in terms of shared mechanisms (Jaeggi et al., 2010, 2014). As such, a key assumption of the training literature is that these kinds of transfer benefits can occur when there is cognitive and/or neural overlap between the processes tapped in both training and transfer, irrespective of domain (Dahlin et al., 2008). This overlap is termed *process-specificity*: if a certain component of EC – e.g., updating– is targeted and improved over the course of training, then transfer tasks that also rely on updating processes should also be affected, even if the task itself appears superficially different (say, in terms of stimuli characteristics).

To inform our discussion, we reviewed the literature on EC interventions in children and adults and then sorted the papers by population (i.e., healthy versus at-risk children), and by mode of outcome measure (behavioral and/or neural). For the selected developmental populations, when necessary, we consulted reviews on each separate topic, integrating relevant information from those reviews into our own.

This paper is organized into two parts. In Part I, we discuss the development and neurobiology of EC, followed by an examination of experiences that can affect the development of EC in both negative (e.g., stress, low-SES) and positive (e.g., schooling, music education, martial arts) ways. We then review the training literature involving “at-risk” groups, focusing particularly on low-SES and ADHD, and the effectiveness of certain kinds of EC interventions, guided by an understanding of the factors that positively influence EC. This field is new, and consequently the results are still inconclusive. Note that for the purpose of this review, we will focus on select developmental groups, and we will not review ongoing EC intervention work that targets older adults or adults with psychopathologies, but rather, we refer the readers to other recent reviews on those topics (Kueider et al., 2012; Vinogradov et al., 2012; Wiers et al., 2013). In Part II, we briefly review the neuroimaging intervention literature in healthy populations to guide future training studies involving populations that are likely to demonstrate poor EC. The early state of this enterprise, however, suggests major inconsistencies: no clear picture emerges in terms of brain activity changes and reorganization post-training. This discrepancy renders it difficult to draw generalizable conclusions, but the field is emerging rapidly, requiring evaluation of the current state of affairs. Moreover, neuroimaging studies of clinical or at-risk groups in the context of intervention research are likely to be even more complicated and problematic, especially when considering atypical behavioral and neural profiles. We therefore suggest some candidate neuroimaging analyses (i.e., connectivity and multivariate approaches) that emphasize the ability to examine spatial and temporal patterns in terms of network dynamics, which can reveal a delicate interplay across brain regions and systems. Such methods have the potential to be more informative relative to traditional univariate approaches that test for pre/post activation differences within cortical patches in isolation.

## PART I

### THE DEVELOPMENT OF EC AND ITS NEURAL SUBSTRATES

Executive control has long been associated with the PFC (Shimamura, 2000; Miller and Cohen, 2001), which is among the last cortical regions to fully develop: EC abilities undergo protracted maturation over the course of childhood and adolescence (Thompson-Schill et al., 2009). Moreover, the literature on neuroanatomical development across the whole brain points to dynamic changes that occur postnatally and throughout childhood, with initially undifferentiated regions becoming increasingly functionally specialized (Oliver et al., 2000). This development can occur at different rates: for instance, frontal brain regions undergo change up to age 25, with some frontotemporal tracts not reaching maturity until age 28. Relatively undifferentiated cortical regions co-occur with earlier (rather than later) development, providing a period in which these undifferentiated neural networks may cover larger areas of cortex. As a result, earlier targeted training might lead to more widespread transfer effects by taking advantage of this undifferentiated stage of cortical development (Wass et al., 2012). Although additional factors – including genetic and environmental predispositions as well as dynamic morphological changes – lead to a complicated interplay of developmental components to be taken into consideration (Scerif, 2010), a younger population that may generally have greater brain plasticity and thus, greater learning potential (e.g., Karmiloff-Smith, 1998; Sonuga-Barke and Halperin, 2010). Primarily for this reason, we focus on EC interventions in developmental populations.

Plasticity accompanies cortical maturation beginning at birth. Humans are born with immature brains, and it has been well established that brain maturation develops throughout childhood and adolescence, with PFC developing last (Sowell et al., 2003; Gogtay et al., 2004). Throughout postnatal development, the neocortex matures through an initial rapid growth process of cell proliferation and changes in synaptic density. During this period, the increase in synaptic connections accompanies dendritic and axonal growth (i.e., fibers for communication that extend from neurons) and myelination (i.e., insulation, thus boosting signal transmission) of the subcortical white matter (Huttenlocher and Dabholkar, 1997). Synaptogenesis is then followed by pruning, a synapse-elimination process that lasts well into the third decade of life (Huttenlocher and Dabholkar, 1997; Petanjek et al., 2011). Critically, these processes dynamically occur at differing rates throughout the brain (Huttenlocher and Dabholkar, 1997). Brain regions subserving sensory functions, such as vision and hearing, develop first, followed by development of temporal and parietal cortices – regions responsible for sensory associations. Higher order cognition areas, such as prefrontal and lateral temporal cortices, which serve to integrate information from primary sensorimotor cortices and modulate other cognitive processes, mature last (Casey et al., 2005b; Petanjek et al., 2011).

Non-invasive neuroimaging technologies have enabled researchers to learn a great deal about the anatomical and functional networks of the developing brain. Early work with positron emission tomography (PET) imaging demonstrated that human PFC metabolizes glucose at a slower rate than occipital, temporal, and parietal cortices (Chugani and Phelps, 1986). There also appears to be evidence for a “fine-tuning” of cortical structures as activation shifts from diffuse to focal recruitment as children develop, with cortical gray matter loss (i.e., a sign of cortical maturation) – occurring last (Brown et al., 2005). Taken together, structural and functional evidence suggests that prefrontal regions associated with integration and goal-directed behaviors mature after regions responsible for primary sensory functions (Casey et al., 2005a), with both progressive and regressive processes (rather than simple linear patterns of change) underlying changes in cognitive abilities (Brown et al., 2005; Amso and Casey, 2006).

Cognitive training studies in children seek to take advantage of this relatively undifferentiated brain state (cf. Wass et al., 2012) to maximize possible transfer before pruning accompanies neural specialization. These interventions can take many forms, including mindfulness training, core or supplemental curricula (see Diamond and Lee, 2011); here, we focus on cognitive interventions that specifically target EC processes. For example, guided practice can improve children’s performance on a dimensional card sorting task, wherein children are given feedback when they perseverate after a dimension switch (Brace et al., 2006). Additional studies have used intervention paradigms in children to improve EC abilities through training (Holmes et al., 2009; Thorell et al., 2009; Jaeggi et al., 2011; Loosli et al., 2012). Still others have sought to improve the symptoms associated with disorders such as developmental dyscalculia (Kucian et al., 2011), reading abilities in at-risk youth (Yamada et al., 2011), and dyslexia (Temple et al., 2003). Such behavioral interventions seek to demonstrate improvement on EC – that is, those skills that are critical for complex cognitive functioning and scholastic achievement (Diamond and Lee, 2011).

Importantly, however, there are arguments for why the costs associated with late PFC development (and consequently, immature EC) may be overshadowed by learning benefits that accompany this slow maturational progression. Specifically, an underdeveloped frontal cortex (i.e., hypofrontality) might confer ways for the developing child to increase uptake of bottom-up regularities, which are important for creativity and language learning tasks (Gleitman et al., 1984; Thompson-Schill et al., 2009; Chrysikou et al., 2011). The general idea is that attending to top-down rules – while good for EC task *performance* – may interfere with important *learning* and classification procedures. For example, Ramscar and Yarlett (2007) demonstrated that children are easily able to master learning how to pluralize irregular nouns (e.g., mouse → mice) rather than adopting a pluralization dictated by the more frequent convention or rule (e.g., mouse → mouses). This result points to the idea that children maximize probabilistic input, which in this case optimizes learning. In contrast, adults tend to monitor for rules and alternative patterns using top-down strategies, which may impair certain aspects of language learning that benefit from bottom-up (data-driven) modes of thinking (Ramscar and Yarlett, 2007). This age-related difference in learning may be promoted by immature EC abilities driven by an underdeveloped PFC. In view of these trade-offs, we believe that future interventions aimed at improving performance in developmental groups must strongly consider whether, on balance, the potential costs to learning will outweigh the potential benefits to performance. We focus on select at-risk groups in this review (rather than typically developing children) because the potential performance benefits of EC interventions may outweigh the learning costs.

In sum, these examples demonstrate that an immature PFC bears negative consequences for cognitive performance in certain tasks, but that it can also provide benefits for learning and creativity. In particular, hypofrontality may confer learning benefits at the expense of performance costs, so interventions geared toward young children should consider this trade-off. Thus, the argument for accelerating maturation of EC networks that are mediated by regions of prefrontal cortices through interventions may need to be tempered with the evolutionary and developmental advantages that immature frontal lobes may confer.

Such critical periods of development suggest that children may be affected by both positive and negative factors during particular time windows that could shape cognition and EC development (Nelson, 2000; Knudsen, 2004). In the following sections, we review some of these factors. We then review the training literature of some “at-risk” groups: as part of this research, the training protocols consider the conditions that favorably affect EC performance, in hopes of offsetting the negative consequences of environmental and biological circumstances that compromise EC.

### FACTORS THAT NEGATIVELY AFFECT EC DEVELOPMENT

#### Socioeconomic status (SES)

Environmental factors, including SES, can significantly affect cognitive and brain development (Noble et al., 2007). SES is a composite measure of economic and non-economic factors, including material wealth, social prestige, and education. Educational advocates have long discussed the negative implications of low-SES backgrounds on cognition and, ultimately, on academic achievement (Sirin, 2005; Duncan and Sojourner, 2013). Thus, negative environmental factors such as SES play a role in shaping candidate neural pathways by which (negative) early life experiences might compromise academic achievement or increase the risk of mental illness (Hackman and Farah, 2009; Hackman et al., 2010). Because neurobiological systems may mediate these SES-cognition gradients, we focus here on research demonstrating a link between SES and assessments of EC. For example, Noble et al. (2005a) found that in kindergarteners differing in SES backgrounds, low-SES children performed worse than middle-SES children on measures of language (mediated by the left perisylvian regions) and EC (mediated by thePFC). The groups did not differ on other cognitive measures, and the authors point to the delayed maturation of the brain regions mediating these abilities as being more susceptible to environmental factors such as SES. A subsequent study of individual differences using a population of first-graders demonstrated that SES explained 30% of the variance in language performance (Noble et al., 2007). SES also explained 6% of the variance in EC performance (despite the small values, they were statistically significant in both cases).

While the role of SES has been examined in sociological and epidemiological contexts, research is just now beginning to shed light on its impact on neurobiological mechanisms. For example, in 5-year-old children, SES predicts hemispheric asymmetry of the inferior frontal gyrus – which is well known to support critical EC functions – even after controlling for scores on a standardized set of language and cognition tests, with left lateralization associated with higher SES (Raizada et al., 2008). Consistent with this result, Sheridan et al. (2012) found that right medial frontal gyrus (rMFG) activity was inversely related to accuracy in acquiring a novel stimulus–response association (e.g., through a dimensional change card sorting task). Finally, cortical thickness measures vary differentially based on individual IQ levels. Not only might prolonged cortical thickening reflect extended synaptogenesis in individuals with high IQ, but this measure is also linked to increased levels of environmental input (Brant et al., 2013).

Additionally, Stevens et al. (2009) have demonstrated that SES differences can contribute to development of neural mechanisms of selective attention by measuring event-related potentials (ERPs), which provide an electrophysiological response to stimuli that is temporally precise. In particular, ERPs can provide an index of selective attention by demonstrating an amplified neural response in the N1 early negative component, which occurs in the first 100 ms after stimulus presentation (Hillyard et al., 1973). In one study, ERPs were measured while children were cued to selectively attend to one sound source and ignore the other. Compared to high-SES children, low-SES children showed reduced effects of selective attention on neural processing, as seen in an altered N1 response over bilateral frontal and central electrodes. Specifically, children in the lower SES group had a larger amplitude response to probes in the *unattended* channel relative to children in the higher SES group, suggesting an impaired ability to suppress a response to distracting information (Stevens et al., 2009). This parallels other ERP work suggesting that low-SES populations have difficulty inhibiting distracting information (D’Angiulli et al., 2008); they also show an attenuated response to novel stimuli relative to high-SES children (Kishiyama et al., 2009).

These findings demonstrate negative behavioral and neural consequences of low SES on EC development, suggesting that this population might be a prime candidate for EC remediation. We return to this issue in the section entitled, “What Groups Might Benefit from an EC Intervention?”

#### Early life stress

Early life stress (ELS) is the exposure to childhood events that challenge a child’s emotional and physical well-being, exceeding their ability to cope with the events (Gunnar and Quevedo, 2007; Pechtel and Pizzagalli, 2010). Some of these stressors can include abuse, neglect, social deprivation, or household dysfunction (Brown et al., 2009). Further, although acute instances of stress can activate the body’s stress response resources in a beneficial manner, high and especially chronic levels of stress can perturb typical brain development (Pechtel and Pizzagalli, 2010).

Neurobiological and neuroendocrine studies suggest that ELS might interfere with typical brain development by accelerating synaptic pruning and aberrantly increasing myelination (Teicher et al., 2006; Paus et al., 2008), although magnetic resonance (MR) neuroimaging techniques do not currently have the resolution to test this empirically (Gogtay and Thompson, 2010). However, studies largely support negative impacts of ELS on cognitive function, which are accompanied by decreased intracranial volume, reduced cross-hemisphere integration, and a smaller corpus callosum (Schiffer et al., 1995; Teicher et al., 2004; Noble et al., 2005b). In line with these findings, microstructural integrity of the corpus callosum may be reduced after ELS exposure (Paul et al., 2008).

In addition to showing effects of ELS on memory (Carrion et al., 2001; Karl et al., 2006) and affective function (Dillon et al., 2009), ELS also impacts EC (Colvert et al., 2008; Bos, 2009; Pollak et al., 2010). Mueller et al. (2010) conducted an functional magnetic resonance imaging (fMRI) study in which adolescents exposed to ELS performed a variant of the go/no-go task involving “go” and “change” trials. Relative to controls, ELS adolescents had longer response times on “change” trials that were accompanied by increased activity in the inferior frontal cortex and striatum. One possible explanation for this group difference is that these frontal regions might be more active to compensate for reduced inhibitory capacity, which has also been observed in women with ELS histories (Navalta et al., 2006).

ELS is correlated with low SES, and both negative factors can spur the development of psychopathologies, including anxiety and attention deficit hyperactivity disorder (Heim and Nemeroff, 2001; Lupien et al., 2009) – two clinical syndromes that we discuss in the section entitled, “What Groups Might Benefit from an EC Intervention?” Generally, this work suggests that ELS and low SES can result in altered prefrontal function, with negative consequences for various cognitive domains, including those subserving EC. Current neuroimaging approaches such as interregional connectivity network analyses might yield a better understanding of the effects of SES and ELS on neurobiology by painting a broader picture of whole brain dynamics. We return to this issue in more depth later in Part II.

### FACTORS THAT POSITIVELY AFFECT EC DEVELOPMENT

Thus far, we have reviewed the ontogeny of EC abilities and the impact that negative factors can have on EC development. However, there is evidence from experiments showing that broad training – outside the lab – can positively affect EC development. These naturalistic interventions can take the form of aerobic exercise and games (Davis et al., 2011), music training (Rauscher et al., 1997; Budde et al., 2008), being raised in a bilingual environment (Calvo and Bialystok, 2014), and yoga (Manjunath and Telles, 2001), providing some evidence for EC improvements, particularly when EC demands are the greatest. Social pretend play, in which children must inhibit acting out of character and flexibly adjust as their friends improvise, improved performance on child versions of the dot-probe and flanker tasks (Diamond et al., 2007).

These various forms of training each have EC components that are central to the tasks – for example, children in martial arts training, including taekwondo, begin each session by directing attention toward themselves (Diamond and Lee, 2011). They monitor, evaluate, and adapt their thoughts and actions, and this practice can lead to increased concentration (Konzak and Boudreau, 1984) and cultivation of mental capacity (Seitz et al., 1990). Lakes and Hoyt (2004) observed children after a 3-month taekwondo intervention, finding that they were more able to focus attention and efforts on the task at hand, and they also improved on an intellectually challenging mathematics task. By engaging in naturalistic forms of training that incorporate EC components, these interventions – might generalize to improvement on other tasks that also involve shared EC abilities. Moreover, the experience of the classroom itself might boost EC abilities beyond natural development. For example, Burrage et al. (2008) demonstrated that early schooling has a significant impact on EC abilities in a group of pre-kindergarten and kindergarten children when compared to children at the same age who did not attend school, even when assessing these abilities before schooling began and when controlling for factors such as SES and race.

In view of these findings, various kinds of interventions might be especially useful for children with poorer EC abilities, in that they may enjoy more benefits from an intervention (Diamond, 2012). Given the association between EC abilities and numerous cognitive skills, academic outcomes, and clinical psychopathologies, targeted EC interventions during childhood may be particularly useful (keeping in mind the caveats outlined earlier related to top-down/bottom-up trade-offs). To this end, we now review specific at-risk groups that could profit from an EC intervention. Although numerous factors might result in an educational achievement gap for these populations, timely educational interventions may be able to minimize or even close this gap by positively impacting EC development.

### WHAT GROUPS MIGHT BENEFIT FROM AN EC INTERVENTION?

#### Low SES

The susceptibility of prefrontal cortices to experiential and environmental factors such as SES and life stress described earlier raises the question of whether these cortical networks are subject to improvement through intervention, as such negative experiences can range widely in scope. In other words, the differences observed in brain and behavioral function in low- relative to high-SES children is experience-dependent, where experience is defined by real-life economic and social circumstances. Here, laboratory-developed interventions might be able to create new experiences for low-SES children to mitigate the gaps outlined above.

In a broad family-based intervention, Neville et al. (2013) developed “Parents and Children Making Connections - Highlighting Attention,” or PCMC-A – a program that combines training sessions for parents with concurrent attention training exercises for children. These exercises are designed to improve regulation of attention and emotion states. Over the course of 8 weeks, low-SES preschoolers who were enrolled in Head Start completed either the PCMC-A program, an active control training program that focused on child classroom training, or remained in Head Start alone (i.e., no supplemental training). After the intervention, PCMC-A children demonstrated improvements of measures of non-verbal IQ, receptive language, and pre-literary skills; furthermore, their parents reported reduced stress levels. Before training, the groups did not show differences in ERP signatures of early attentional modulation to either attended or unattended stimuli, suggesting an inability to shift attention toward either sound source. However, after the intervention, only the ERP signatures of the PCMC-A group demonstrated improved selective attentional processing as a function of the intervention. Along with another study demonstrating that low-SES children may profit from targeted EC interventions (Goldin et al., 2014), these early results hold promise for using interventions to target at-risk children and may serve as precursors to subsequent behavioral effects.

Other types of targeted interventions may be able to counteract the negative consequences of environmental factors such as SES on distinct cognitive processes. In one study by Mackey et al. (2011), low-SES children trained for 8 weeks on either reasoning or speed processing using a battery of commercially available games. After training, reasoning-trained children completed more matrix reasoning problems, and speed-trained children improved significantly on a measure of cognitive speed that requires rapidly translating digits into symbols. Finally, although the reasoning-trained group also showed improved measures of spatial WM span, these gains did not appear to be related to reasoning gains. Taken together, the results suggest that both cognitive processes – reasoning and speed – are separately modifiable by targeted interventions, and that these improvements are seen in a special low-SES population that may need the intervention more than others in order to reduce the achievement gap (Mackey et al., 2011). Although the neural correlates of these interventions have not yet been tested, one possibility is that the interventions may alter the rate of white matter maturation, wherein the degree of white matter development influences processing speed, which might in turn support improved reasoning ability (Ferrer et al., 2013).

In sum, behavioral work is beginning to shed light on how both broad and targeted interventions can positively impact EC abilities in low-SES populations, but more work will be necessary to better understand the neurobiological changes underlying improved EC as well as the time-course for these changes. We will again return to these ideas in Part II.

#### Attention deficit hyperactivity disorder (ADHD)

We have described some research showing that children – a population with late-developing EC abilities, partially due to immature neural “hardware” – might benefit from EC interventions that specifically target these abilities, yielding improvement on untrained tasks (at least in the lab). Intervention work may also benefit other groups who demonstrate poor EC skills relative to adults and their non-clinical counterparts, for example, certain clinical populations that are prevalent in a child population (e.g., ADHD). Similar to low-SES groups and in contrast to healthy populations, the impaired EC abilities in this clinical syndrome might make these children prime candidates for EC remediation. For this reason, we turn now to work that has examined this possibility in ADHD.

ADHD affects 3–10% of children in the United States (e.g., Merikangas et al., 2010) and is defined by inattention, impulsivity, and hyperactivity with broad deficits in EC (Barkley, 1997). ADHD, linked to impaired function of the frontal lobes (Castellanos and Proal, 2012), can negatively impact educational achievement, job success, and social well-being (Kessler et al., 2006; Loe and Feldman, 2007). The prevalence and impact of ADHD in children has led researchers to implement cognitive interventions to help this population. For example, an early study used an adaptive (i.e., adjusting for difficulty as the participant’s performance improved) and intense (i.e., repeated several times a week for at least 5 weeks) intervention in ADHD children. In addition to improving on the trained WM task, participants significantly improved on an untrained WM task, as well as on Raven’s Progressive Matrices (RPM), a non-verbal complex reasoning task (Klingberg et al., 2002). Using Cogmed training, one study demonstrated that across measures of WM, inhibitory control, and complex reasoning, ADHD children who trained on Cogmed outperformed those children completing a control training program (Klingberg et al., 2005). There are also indications that WM training may alter academic performance in these populations (Green et al., 2012), and some of these effects can persist for months after the end of training, suggesting that long-term changes are possible with short, intense training periods.

Despite the initial promising effects of the Cogmed WM training program, more recent studies using Cogmed in children with ADHD have been mixed (Chacko et al., 2013). For example, two studies showed improvements to neuropsychological outcomes and parent-rated ADHD symptoms relative to both wait-list control and placebo treatment conditions (Klingberg et al., 2005; Beck et al., 2010). However, a third study did find improvements to behavioral observation during an academic task but no improvements in parent-rated ADHD symptoms (Green et al., 2012). A fourth study using an active-control group found no group differences between the training and control groups (Gray et al., 2012). Other approaches have used forms of computerized attention training: training on sustained, selective, alternating, and divided attention using visual and auditory stimuli (Shalev et al., 2007). In a separate study, relative to a no-contact control group, the researchers found small to moderate improvements on EC measures of inhibition, planning, comprehension and memory of verbal instructions, and cognitive flexibility. However, these small effects were also accompanied by several transfer measures that did not reveal any significant effects at all, and the evaluators were not blind to each participant’s assigned condition (Tamm et al., 2013). Some of these behavioral study limitations and discrepancies may be attributed to the lack of alignment between treatment outcomes and the model of therapeutic benefit, a lack of theory-driven overlap between the training regimen and outcome measures (e.g., in terms of cognitive mechanisms tapped; see Dahlin et al., 2008), the equivalence of the control conditions, and examining the individual differences in treatment response (Shah et al., 2012).

In a pair of studies that combined behavioral WM training with neuroimaging in order to uncover neural changes in the ADHD population after an intervention, Hoekzema et al. (2010, 2011) observed functional and structural changes after training ADHD children on tasks tapping WM, cognitive flexibility, attention, planning, and problem solving. Functionally, during inhibition, researchers observed increased activation in orbitofrontal cortex, superior frontal cortex, middle frontal gyrus, and inferior frontal cortex. Performance during attention tasks was associated with increased cerebellar activity (Hoekzema et al., 2010). Structurally, the researchers observed volumetric gray matter increases in bilateral middle frontal cortex and right inferior–posterior cerebellum after training compared to controls. Furthermore, the extent of gray matter volume increase in cerebellum was associated with attentional performance. Interestingly, the regions demonstrating training-related changes are some of the same regions that are typically characterized by volume *reduction* in ADHD patients. If these regions subserve ADHD behavior, then cognitive training might counteract some of the neuroanatomical reductions associated with the disorder and its symptoms (Hoekzema et al., 2011), with cognitive training playing a functionally restorative role that ultimately leads to compensatory increased gray matter volume.

To conclude, although promising, EC intervention work targeting ADHD is still in its early stages and the findings are still too varied to warrant strong conclusions about positive effects. Some of the current behavioral and neurobiological limitations for this line of research might be addressed by broadening the scope and procedures of the training, as well as by embracing an interdisciplinary approach that can better conceptualize and enhance cognitive training in ADHD as a possible therapeutic target (Rutledge et al., 2012). This work may also generally benefit from novel neuroimaging techniques that can more comprehensively assess spatial and temporal brain changes that yield (and inform an interpretation of) behavioral improvements.

## PART II

In Part I of this review, we discussed how EC ontogeny and negative factors impacting its development might be mitigated by interventions that target EC abilities, and we described several populations who might be good candidates to focus training efforts because of the educational, economic, and social implications of poor EC. However, we do not yet have a clear understanding of the neurobiological changes that induce behavioral improvements following an EC intervention. Indeed, in some cases, we also lack a clear understanding of the cognitive mechanisms that are trained during a regimen and are common to the outcome measures to effect transfer. The behavioral work that we described previously raises particular questions about the spatial profile and time-course of the neurobiological changes. In other words, it remains untested what brain systems underlie transfer effects in special groups because the neuroimaging component of EC interventions in these populations is in a relative phase of infancy. Such research is critical to conduct to (a) validate behavioral effects (e.g., by examining common brain-behavior changes post-intervention) and (b) use as a precursor to behavioral effects that have not emerged. Namely, can structural and/or functional brain activity patterns predict who within a special group is likely to benefit from training? The answer to this question could shed light on some of the mixed findings reviewed earlier.

In theory, meanwhile, studies of EC interventions in healthy populations could inform which neurobiological (and cognitive) mechanisms should be targeted in special groups in hopes of maximizing transfer success. Given some inconclusive findings reviewed in Part I, the time is ripe to consider this issue. It is widely accepted that for routine practice with a training task to confer transfer benefits to an (unpracticed) outcome measure, some underlying cognitive and neural processes must be shared across both tasks (Dahlin et al., 2008). For example, Dahlin et al. (2008) demonstrated that after 5 weeks of memory-updating training, behavioral improvements transferred to a WM 3-back updating task but not to a Stroop task. Critically, both the updating task and the WM task engaged the striatum, whereas the Stroop task―a prefrontal conflict resolution task―did not. These results provide evidence that shared neural substrates underlie process-specificity: the idea that transfer can occur when both training and transfer tasks recruit overlapping processing and neurobiological components.

We discussed earlier that EC comprises multiple components and is not, rather, a unitary construct (Friedman and Miyake, 2004). In view of this, it may be unsurprising that in studies of healthy adults, many EC interventions do not result in widespread transfer (Morrison and Chein, 2010; Hindin and Zelinski, 2012; Redick et al., 2012; Melby-Lervåg and Hulme, 2013; Sprenger et al., 2013; Thompson et al., 2013), perhaps due to a weak link between the components tapped during training and those tapped in the outcome task(s) (Jaeggi et al., 2010). A similar argument could plausibly explain some of the unreliable results described earlier in special groups. A process-specificity framework however might afford some traction in the future, particularly in special groups, to better understand the mixed results outlined in Part I. Although not the focus of those studies, the use of neuroimaging methods to evaluate common neurobiological structures and cognitive procedures could inform what range of processes are affected in clinical groups and thus what ought to be the focus of training. Moreover, because the neural mechanisms underlying a complex set of behavioral changes in these select groups is so poorly understood, a process-specific approach could also help to generate testable predictions by providing a candidate set of neural networks on which to focus analyses. This suggestion follows the tradition of lesion-deficit analyses in neuropsychological groups (e.g., aphasics), where mapping specific symptoms associated with a complex syndrome (rather than an entire syndrome itself) onto specific brain structures has been a more fruitful approach (e.g., Dronkers, 1996; Robinson et al., 2005), as attempts to localize multifaceted disorders in the brain has yielded little consistency. Rather, the various symptoms associated with a complex syndrome typically reveal an intricate network of involved regions, and process-specific contributions to a network could provide insight into which parts of a network might change depending on what the target is of a particular intervention.

Non-invasive brain imaging is a valuable method for examining the neural mechanisms that underlie the observed behavioral changes in EC resulting from intervention. By shedding light on some of the cortical mechanisms involved in the training and transfer tasks (see **Figure 1**), it provides potential explanations for the brain-behavior relations that give rise to transfer benefits. It might also provide information on why transfer does not occur in some circumstances. In what follows, we review some research that has investigated brain activity changes in healthy groups in the context of EC intervention. To preview, although there are intriguing and interpretable trends within any given study, there is a fair amount of inconsistency in the findings across studies, thereby preventing a clear and uniform description of what happens neurobiologically following intervention. This result is somewhat problematic for a broad understanding of process-specificity, as it might apply to EC interventions for the special groups outlined in Part I. We suggest that one issue is that most studies of this sort focus analyses on brain changes in isolation, rather than on dynamic changes in a networked system of brain regions acting in concert. This limitation makes the current state of the science difficult to forge obvious paths for proceeding with special groups. We therefore conclude with some ideas about other analysis techniques that are designed to evaluate brain-network dynamics that could ultimately bypass current limitations and offer a better, more comprehensive understanding of the advantages and constraints of EC intervention.

### NEUROIMAGING OF INTERVENTION WORK IN HEALTHY POPULATIONS

Neural changes that accompany behavioral results following training can take a number of forms; we focus here on functional rather than structural changes (Kelly et al., 2006; Buschkuehl et al., 2012). Using fMRI, some researchers have hypothesized that training should increase neural activation magnitude, because this direction of the effect is thought to reflect neural strengthening following practice (i.e., better behavior = more neural recruitment). Some studies have indeed reported increases in the functional activation of brain regions recruited during training (Temple et al., 2003; Shaywitz et al., 2004; Stevens et al., 2008; Hoekzema et al., 2010; Jolles and Crone, 2012). For example, Olesen et al. (2004) found WM-related activity increases in the middle frontal gyrus and parietal cortex after 5 weeks of WM training, replicating some of these results at the single-subject level (Westerberg and Klingberg, 2007). Another study trained young adults on forwards and backwards object span for 6 weeks, which resulted in increased activation in default-mode regions during the forward condition, accompanied by increased activation in the striatum and left ventrolateral PFC in the backward condition (Jolles et al., 2011). Finally, Buschkuehl et al. (2014) found increased perfusion in frontal and occipital regions as a function of a short (one-week) intervention. These activation increases might be attributed to an increase in the size of training-related cortical representations over time, or to a strengthened neural response from brain areas already active pre-training (Pascual-Leone et al., 2005; Kelly et al., 2006).

In contrast, other researchers have hypothesized that training should result in decreases in neural activation magnitude (Qin et al., 2004; Haier et al., 2009; Kucian et al., 2011), which could be attributed to increased neural efficiency that develops over the course of the intervention period (Kelly et al., 2006; Neubauer and Fink, 2009; Nyberg et al., 2009). Using an adaptive WM intervention with the n-back task, Schneiders et al. (2011) found that after training, participants showed decreased activation in the right superior middle frontal gyrus and posterior parietal regions. Another study scanned participants three times over the course of 4 weeks while they practiced the n-back task: although the researchers initially observed increased activity in the intraparietal sulcus and superior parietal lobe midway through their training protocol, these same regions demonstrated decreased activation at the end of their training protocol (Hempel et al., 2004).

Other researchers have suggested that training can result in neural re-distribution, namely, a combination of activation increases and decreases. For example, Dahlin et al. (2008) found increased activity in the striatum after participants trained for 5 weeks on an updating task, which was accompanied by activation decreases in frontal and parietal regions. In the same paper described earlier, Olesen et al. (2004) conducted a second experiment in which they trained young adults on visuospatial WM tasks for 3 weeks. After training, they found increased activation in frontal and parietal regions, as well as the basal ganglia and thalamus; this effect was accompanied by activation decreases in the anterior cingulate, post-central gyrus, and inferior frontal sulcus. In all of these studies, the decreased activation in regions that mediate attentional control might reflect a shift from sustained attention required to perform a novel task toward more automated processing. The increased activation might reflect increased neural strengthening following practice (akin to Hebbian learning principles of neuronal firing).

These mixed results make it difficult to draw definitive conclusions about the impact of training on brain function and call for neuroimaging techniques that can effectively assess dynamic neural changes over time. However, one hypothesis to explain the variation above is that training time (or task proficiency) may drive the neural changes observed after training. Specifically, short periods of training may increase neural activation―reflecting increased effort in learning and adapting to task demands―whereas progressively expert task proficiency in carrying out a particular cognitive function may result in decreased activation (reflecting increased neural efficiency). Emerging techniques in neuroimaging (e.g., network analyses) are attractive methods for testing this hypothesis because changes in network functions may be able to reveal a dynamic interplay among regions that underlie plasticity.

### CURRENT AND FUTURE DIRECTIONS

#### Connectivity analyses

**FIGURE 2:**
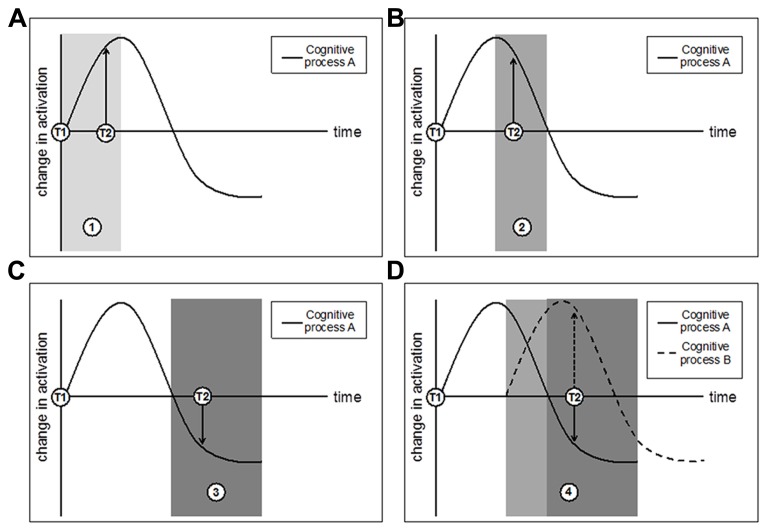
**A hypothetical network trajectory for changes in brain activation as a function of training, where T1 indicates pretest and T2 indicates posttest. (A)** T2 taken during time period 1 (lightest gray) will observe an increase in activation that could reflect neural strengthening; **(B)** T2 taken during time period 2 (dark gray) will also observe an increase in activation that is actually tied to a trending decrease in activation; **(C)** T2 taken during time period 3 (darkest gray) will observe a decrease in activation that could reflect increased neural efficiency. Panels **(A–C)** consider a single cognitive process, but important information about network connectivity and co-variation could emerge by considering multiple cognitive processes in concert with one another. **(D)** Multiple cognitive processes with different time-course trajectories may demonstrate a more complex pattern of network changes that are best detected through approaches that can assess changes in cognitive processes *in addition* to their relational co-variation and connectivity. Finally, note that these outcomes do not preclude the potential benefit of multiple interim assessment scans throughout training (which, while informative, may not always be feasible for logistical and financial reasons).

Localization studies―like those described in the previous section―are useful in revealing the spatial profile of isolated brain activity of particular regions but may not yield the most comprehensive or consistent picture of neural dynamics. Moreover, it is clear that brain regions form networks for communication rather than act exclusively. Connectivity analyses assess these network dynamics, and functional connectivity analyses test the correlation of brain activity across regions that are cooperatively recruited by some mental procedure. Specifically, interregional connectivity data reveal the extent to which activity in one brain area co-varies with activation in other brain areas. This analysis approach can be particularly informative because it paints a broader picture of neural dynamics―at the network level, and beyond that of activity magnitude changes―that can be altered by an intervention. Another benefit is that regional co-variation can yield insight into both process-specificity (brain areas that co-engage during a particular cognitive function) and domain-generality (brain areas that may co-engage with a specific cognitive procedure during some epoch but with another procedure during another epoch, influenced by task demands; see Federenko and Thompson-Schill, 2014). Therefore, network approaches might provide insight into the variable nature of the functional findings described above. For instance, functional connectivity analyses permit researchers to address these questions: What regions of the network change over time, and how do they change? What regions remain activation-stable despite other parts of the network showing activation-variance following intervention? At what point does the initial “ramp up” (reflected by activation increases) that corresponds to the effort associated with the novelty of a training-task procedure become less effortful, more efficient, and automatic (reflected by activation decreases)? Does such “ramp down” occur alongside behavioral improvements on the training task as well as transfer measures? Does it occur together with ramp-up elsewhere in the network, assuming that multiple cognitive procedures are tapped during training? **Figure 2** sketches some hypothetical outcomes of a connectivity approach to training.

Further, the connectivity approach allows researchers to consider intrinsic brain activity at rest in addition to task-related activity, and any differences in resting state connectivity after the intervention could suggest generalized effects beyond that of training task performance. Connectivity analyses can additionally converge with task-related analyses to provide meaningful information about neural changes after interventions (Buschkuehl et al., 2014). Such dynamic interplay among regions could reveal important insights into brain plasticity, which localization approaches might inherently miss.

A few studies have demonstrated increased functional connectivity following training. After a period of intensive reasoning training (i.e., an Law School Admission Test preparation course), Mackey et al. (2013) found that students who had completed the course showed strengthened fronto-parietal and parietal-striatal connections. Moreover, left rostro-lateral PFC had increased resting-state functional connectivity with parietal regions (both within the left hemisphere and between hemispheres). Increased functional connectivity in terms of efficiency (i.e., the extent to which a region connects with other regions) and degree value (i.e., the number of connections that a region has to other network regions) has also been linked to meditation training (Xue et al., 2011). Specifically, the left ACC – a key regional node in the self-regulatory network whose function may serve cognitive as well as socio-emotional purposes (Kelly et al., 2008)―demonstrated increased connectivity after a period of integrative body-mind (or meditation-type) training. Finally, after training participants on an adaptive verbal processing speed task, Takeuchi and colleagues (Takeuchi et al., 2011) observed increased functional connectivity between the left perisylvian area and regions extending to the lingual and calcarine cortex. This increased connectivity might reflect increased verbal information transfer between the regions, and was correlated with behavioral improvements on the processing speed task.

Other studies have seen a combination of increases and decreases in connectivity after an intervention (cf. **Figure 2**). For example, in the same study by Jolles et al. (2011) that found increased activation in the striatum and PFC after 6 weeks of verbal WM training, the researchers also observed increased functional connectivity between the rMFG and other regions of a fronto-parietal network, including bilateral superior frontal gyrus, paracingulate gyrus, and ACC. Further, the degree of increased functional connectivity positively correlated with behavioral performance increases. These connectivity increases were accompanied by decreased functional connectivity between the medial PFC and the right posterior middle temporal gyrus. One potential explanation for these effects could be that the connectivity between these regions reflects both reactive task engagement as well as expectation about co-activation in the future (Körding and Wolpert, 2006; Bar, 2007; Raichle, 2010). In a second study, Takeuchi and colleagues (Takeuchi et al., 2013) administered 4 weeks of an adaptive WM training task, finding that WM-trained participants showed increased functional connectivity between mPFC and the precuneus (both regions that are part of the default mode network, or DMN), as well as decreased connectivity between mPFC and right posterior parietal cortex and right lateral PFC (nodes of the executive attention system, or EAS). The authors argue that these results reflect a shift from the EAS network (activated during task engagement) toward the more automated DMN, which tends to be activated in a task-independent manner (Chein and Schneider, 2005).

In sum, studies that examine connectivity changes – and more generally, analyses that consider network activation and co-variation – may be able to clarify some of the field’s mixed results by providing a broader picture of the neural dynamics that accompany behavioral training and transfer. They may be able to do this because they can assess connectivity measures that account for changes in spatial activation, time-course differences, and network and regional communication and co-activation. In particular, they could shed light on the idea that with practice, EC processes will become more automatic over time, requiring fewer cognitive and neural resources (Chein and Schneider, 2005). For example, one could test this prediction by examining the extent to which connectivity changes in regions that subserve EC components (even if activity decreases) after an intervention, and whether the degree of these changes can be predictive of behavioral performance after training as well. Within a process-specificity framework, one might further predict connectivity changes that are dependent on both training duration and the extent of overlap across cognitive processes (i.e., the shift in distance between distinct cognitive processes – see **Figure 2D**).

#### Multi-voxel pattern analyses

Activation increases and decreases―such as those described previously―are usually based on general linear modeling (GLM) analyses of neuroimaging data. Each volumetric unit of the imaged brain (usually termed a “voxel”) carries a time-series of information, with approximately 40,000 data-points (i.e., one for each voxel in the brain) collected every few seconds over the course of an experiment. In fMRI, the GLM approach―which is the standard in the field―involves analyzing the information from these voxels to separate stimulus-induced signals from noise. However, this modeling approach comes with a set of assumptions that, when considering neurobiological mechanisms more naturalistically, may become limitations (for a comprehensive review, see Monti, 2011). Specifically, GLM approaches assume that the activity in each voxel of the brain occurs independently from every other voxel in the brain. While the assumption is more likely to be true for voxels that are located far apart from one other, its validity is somewhat more limited when considering two voxels that sit near or adjacent to one another. In these cases, the GLM approach may not fully capture spatially distributed information that goes beyond meaningful signal in individual voxels.

Thus, analysis approaches that consider meaningful patterns of activation, rather than a set of independently activated voxels, might yield informative results with important implications. This kind of “pattern-analysis” approach, often termed multi voxel pattern analysis (MVPA), considers patterns of activation rather than individual voxels, and therefore carries the additional benefit of not having to rely solely on voxel-by-voxel activation. Moreover, the high spatial frequency information detected by MVPA is conducive to performing within-subject analyses. That is, traditional GLM analyses necessarily average activity across subjects’ brains (which vary wildly in terms of size, morphology, and location of particular regions), and patterns in one individual may not generalize to others. An MVPA approach, in contrast, affords greater sensitivity toward detecting neural patterns, and thus has the potential to identify information about brain activation patterns within individual subjects and cater to individual differences in these activation patterns. MVPA has primarily been applied in the long-term memory and visual domains. For example, it has been used to predict recall of object categories (Polyn et al., 2005), demonstrate distributed neural representations of objects (Haxby et al., 2001), decode brain states during near-threshold fear detection (Pessoa and Padmala, 2006), distinguish between lexical and syntactic neural information (Fedorenko et al., 2012), and link behavioral and neural measures of conceptual similarity (Weber et al., 2009). To our knowledge, MVPA has not yet been applied to the neuroimaging of EC training studies, but might reveal subject-specific brain states that are predictive of behavioral changes following an intervention.

For example, the degree of similarity between brain activation patterns before and after training may predict the extent of observed behavioral improvement on the training tasks; additionally, similarity between these training patterns and brain patterns associated with outcome measures might be indicative of transfer success (or at least, a precursor to it). Both of these measures could ground MVPA findings within a process-specificity framework by providing a concrete measure of similarity between training and transfer brain activation patterns. Further, by being able to cater to single subjects and account for individual differences that may be masked through a group-level analysis, it may uncover neural mechanisms underlying some of the individual differences in behavioral training and transfer. Thus, its analytic appeal might make it an attractive analysis candidate for future intervention work.

## CONCLUSION AND CLOSING REMARKS

In this review, we have discussed several examples of populations for which training EC might serve as a useful intervention strategy, as well as how emerging neuroimaging techniques might inform the mixed results from these groups.

Though much work has been devoted to the *behavioral* transfer effects of training, some information on the *neural* transfer effects of training in healthy adults is beginning to emerge. The neuroimaging intervention work in healthy populations, as a result, may be able to inform future work on post-intervention neural changes in select developmental and at-risk populations, although this field is relatively young and thus faces challenges. Further, we have suggested some possible neuroimaging analysis techniques – namely, connectivity of neural networks and multivariate pattern analysis – that might provide additional guidance by examining brain states as well as intra- and inter-regional connectivity patterns before and after training.

We have discussed a few selected groups whose relatively poor EC skills make them prime candidates for EC intervention, but the current results from that work are mixed. A process-specific account – not usually the focus of intervention work in these populations – could be informative in both better understanding when transfer does and does not occur, and helping to guide future neuroimaging work in this field. Emerging neuroimaging approaches – namely, connectivity and MVPA analyses – may also be able to paint a more comprehensive picture of the undoubtedly complex neural profiles in these groups (and their potential plasticity). Finally, additional research on the basic science mechanisms underlying EC training could have important social, educational, and economic implications as it works to guide and inform future training paradigms targeted toward specific populations.

## AUTHOR CONTRIBUTIONS

Nina S. Hsu, Jared M. Novick, and Susanne M. Jaeggi wrote the paper.

## Conflict of Interest Statement

The authors declare that the research was conducted in the absence of any commercial or financial relationships that could be construed as a potential conflict of interest.
